# Dietary induction of NQO1 increases the antitumour activity of mitomycin C in human colon tumours *in vivo*

**DOI:** 10.1038/sj.bjc.6602171

**Published:** 2004-10-05

**Authors:** A Begleiter, M K Leith, J A Thliveris, T Digby

**Affiliations:** 1 Department of Internal Medicine, Manitoba Institute of Cell Biology, CancerCare Manitoba, University of Manitoba, 675 McDermot Avenue, Winnipeg, Manitoba, Canada R3E 0V9; 2 Department of Pharmacology and Therapeutics, Manitoba Institute of Cell Biology, CancerCare Manitoba, University of Manitoba, 675 McDermot Avenue, Winnipeg, Manitoba, Canada R3E 0V9; 3 Department of Human Anatomy and Cell Science, University of Manitoba, 730 William Avenue, Winnipeg, Manitoba, Canada R3E 3J7

**Keywords:** mitomycin C, antitumour activity, NQO1, diet, induction

## Abstract

The bioreductive antitumour agent, mitomycin C (MMC), requires activation by reductive enzymes like NAD(P)H:quinone oxidoreductase 1 (NQO1). We used a novel approach to increase MMC efficacy by selectively inducing NQO1 in tumour cells *in vivo*. CD-1 nude mice were implanted with HCT116 cells, and fed control diet or diet containing 0.3% of the NQO1 inducer, dimethyl fumarate (DMF). The mice were then treated with saline, 2.0, 3.5 or 2.0 mg kg^−1^ MMC and dicoumarol, an NQO1 inhibitor. The DMF diet increased NQO1 activity by 2.5-fold in the tumours, but had no effect in marrow cells. Mice given control diet/2.0 mg kg^−1^ MMC had tumours with the same volume as control mice; however, mice given DMF diet/2.0 mg kg^−1^ MMC had significantly smaller tumours. Tumour volumes in mice given DMF/2.0 mg kg^−1^ MMC were similar to those in mice given control diet/3.5 mg kg^−1^ MMC. Tumour inhibition was partially reversed in mice given DMF/2.0 mg kg^−1^ MMC and dicoumarol. DMF diet/2.0 mg kg^−1^ MMC treatment did not increase myelosuppression and did not produce any organ toxicity. These results provide strong evidence that dietary inducers of NQO1 can increase the antitumour activity of bioreductive agents like MMC without increasing toxicity.

Bioreductive agents are a class of anticancer drugs that must be activated in cells by reduction. These agents are preferentially active in solid tumours, which represent the majority of cancers and the highest mortality. The clinical potential (Workman and Stratford, 1993) and mode of action (Rauth *et al*, 1993; Rockwell *et al*, 1993) of these agents have been actively studied, and the use of the prototype drug mitomycin C (MMC) in the clinic was recently reviewed (Begleiter, 2000). Bioreductive agents are generally activated by one-electron reducing enzymes like NADPH:cytochrome *P*450 reductase (RED) (Pan *et al*, 1984; Rockwell *et al*, 1993) or two-electron reducing enzymes like NAD(P)H:quinone oxidoreductase 1 (NQO1; DT-diaphorase) (Riley and Workman, 1992; Ross *et al*, 1993). RED and NQO1 are the major activators for current agents (Begleiter *et al*, 1989, 1992; Hoban *et al*, 1990; Riley and Workman, 1992; Begleiter and Leith, 1993; Ross *et al*, 1993; Patterson *et al*, 1995), but their role varies with the agent, enzyme, oxygen level (Marshall *et al*, 1989; Hoban *et al*, 1990; Begleiter *et al*, 1992) and pH (Siegel *et al*, 1990; Begleiter and Leith, 1993). Reduction of MMC activates alkylating groups that form DNA crosslinks, and this is the most important cytotoxic mechanism for this agent (Lown *et al*, 1976; Pan *et al*, 1986; Tomasz *et al*, 1987; Riley and Workman, 1992). However, in the presence of oxygen, redox cycling occurs to form reactive oxygen species, which can also contribute to cytotoxic activity (Lown *et al*, 1976; Dusre *et al*, 1989; Riley and Workman, 1992).

NQO1 catalyses two-electron reduction of quinones and nitrogen-oxides (Ernster, 1987; Riley and Workman, 1992). Several human diaphorases are known (Jaiswal *et al*, 1990, 1991; Riley and Workman, 1992), but NQO1 is most important for activating bioreductive agents (Jaiswal, 1991; Riley and Workman, 1992; Belinsky and Jaiswal, 1993). NQO1 is a homodimer that uses NAD(P)H as an electron donor (Riley and Workman, 1992). The enzyme is mainly cytosolic, but 5–10% is membrane bound (Riley and Workman, 1992). It is ubiquitous in eukaryotes but levels vary in different tissues (Schlager and Powis, 1990; Riley and Workman, 1992; Belinsky and Jaiswal, 1993), with low levels in haematopoetic cells (Schlager and Powis, 1990; Siegel *et al*, 2001). NQO1 activity is usually higher in tumour than normal cells, but is lower *in vivo* than *in vitro* (Schlager and Powis, 1990; Riley and Workman, 1992; Belinsky and Jaiswal, 1993; Ross *et al*, 1994; Collard *et al*, 1995). Expression of NQO1 is transcriptionally regulated (Riley and Workman, 1992), and the enzyme is highly inducible by a wide variety of inducers (Prestera *et al*, 1993). The induction pathway is unknown but may involve a cytosolic redox signal that alters expression and/or interaction of transcriptional factors like Jun, Nrf, Maf, Fos and Fra with the xenobiotic response element and the antioxidant response element (Belinsky and Jaiswal, 1993; Favreau and Pickett, 1993; Venugopal and Jaiswal, 1996, 1998; Yao *et al*, 1996; Kepa and Ross, 1999; Nguyen *et al*, 2000). An NF-*κ*B element may also be involved in induction (Yao and O'Dwyer, 1995). NQO1 is induced by a wide variety of dietary and synthetic agents including: dithiolethiones like Oltipraz, an antiparasitic agent; isothiocyanates like sulforaphane, found in cruciferous vegetables, and dietary metabolites like dimethyl fumarate (DMF), a metabolite of fumaric acid which is found in fruits and vegetables (Talalay *et al*, 1988; Prestera *et al*, 1993). NQO1 is a member of the phase II detoxifying enzymes that help to remove xenobiotics from cells and are important in early defence against carcinogenesis (Prestera *et al*, 1993). Inducers of NQO1 like Oltipraz have been tested as cancer preventive agents in animals and humans (Egner *et al*, 1994; Hecht, 1998; Kensler *et al*, 1998).

Activation of MMC by NQO1 has been extensively studied (Riley and Workman, 1992; Ross *et al*, 1993). Cells with elevated NQO1 levels are more sensitive to MMC and drug activity is decreased by the NQO1 inhibitor, dicoumarol (DIC) (Begleiter *et al*, 1989; Marshall *et al*, 1989; Siegel *et al*, 1990). NQO1 is a major activating enzyme for MMC and other bioreductive agents including, RH1 and MeDZQ (Workman and Stratford, 1993; Riley and Workman, 1992; Beall *et al*, 1995; Winski *et al*, 1998). Thus, selectively increasing the level of NQO1 in tumour cells by gene transfer (Rauth *et al*, 1998) or by selective induction of NQO1 in tumours may be useful for enhancing the efficacy of bioreductive agents. We showed previously that we could selectively increase NQO1 activity in tumour cells compared with normal cells and that this enhanced MMC antitumour activity *in vitro* (Doherty *et al*, 1998; Wang *et al*, 1999). In this study we investigated whether we could selectively induce NQO1 activity in human tumours implanted in a nude mouse model, and if this would increase the antitumour activity of bioreductive agents without increasing the toxicity of these agents.

## MATERIALS AND METHODS

### Materials

The human colon carcinoma HCT116 cell line was obtained from ATCC (Manassas, VA, USA) and DMEM/Hams F12 media, foetal bovine sera and Hanks balanced salt solution (HBSS) were obtained from Invitrogen (Burlington, ON, Canada). The HCT116 cells tested negative for mycoplasma contamination. DMF, MMC and DIC were from Sigma-Aldrich Canada (Oakville, ON, Canada) as were all reagents for the NQO1 activity assay. NycoPrep 1.077A was from Cedarlane (Hornby, ON, Canada). Bio-Rad DC Protein kit was from Bio-Rad (Canada) Ltd (Mississauga, ON, Canada).

Female CD-1 and CD-1 nude mice (6–8 weeks of age) were obtained from Charles River Canada (Montreal, QC, Canada) and were maintained according to institutional regulations. The mice were fed standard irradiated rodent chow except during the experimental diet period. The experimental diet for the mice was a custom powdered autoclavable, semipurified diet with antioxidant free corn oil and vitamin K instead of menadione from ICN Biochemical Division (Aurora, OH, USA). Diets with different concentrations of DMF in the diet were made by the addition of finely ground DMF to the custom diet followed by autoclaving.

Microvettes CB 300 with potassium EDTA, used for blood collection, were obtained from Sarstedt (St Leonard, QC, Canada). Zap-oglobin II lytic reagent and Isoton II were obtained from Beckman Coulter (Mississauga, ON, Canada). Ketalean and Rompum were obtained from Central Animal Care, University of Manitoba (Winnipeg, MB, Canada).

### Cell lines and clonogenic assays

HCT116 cells were grown in DMEM/Hams F12 media and 10% foetal bovine sera. Clonogenic assays were performed as previously described (Begleiter and Leith, 1993). Cloning efficiency ranged from 51 to 90%. Cells were incubated at 37°C for 48 h in the presence or absence of 5 *μ*M DMF and then were treated with various concentrations of MMC for 1 h. Cells were plated and colonies were counted 6 days later. A linear regression analysis of each concentration–survival curve was obtained and the D_10_ was derived from the negative reciprocal of the regression slope (Begleiter *et al*, 1992). The cytotoxicities were compared statistically by a *t*-test comparing the significance of the differences of the slopes of the concentration–survival curves.

For implantation into CD-1 nude mice, the HCT116 cells were counted, washed three times in HBSS, assessed for viability with trypan blue and resuspended at a concentration of 5 × 10^7^ viable cells per ml in HBSS; 100 *μ*l of the cell suspension was injected subcutaneously into the right flank of each mouse.

### NQO1 activity measurements

NQO1 activity was measured as described previously (Doherty *et al*, 1998) using menadione as electron acceptor and is reported as nmol MTT reduced min^−1^ mg protein^−1^. Protein was determined with the Bio-Rad DC kit using gamma-globulin as protein standard. NQO1 activity was measured in 0.25 M sucrose sonicates of HCT116 cells, mouse marrow cells and in sonicates of homogenised mouse tissue. Mouse organs were excised and stored frozen at −80°C in sucrose until analysis. Marrow was obtained by flushing the mouse femurs with HBSS and pooled from several mice. The marrow cells were layered over NycoPrep 1.077A and spun at 600 g for 15 min; the resulting layer of cells was washed and frozen at −80°C in sucrose until analysis. NQO1 activities in HCT116 cells *in vitro* were compared statistically by *t*-test. In mouse organs, the NQO1 activities were compared statistically by Mann–Whitney rank sum test.

### Effect of dose and schedule of DMF on induction of NQO1 *in vivo*

To determine the optimal concentration of DMF in the diet for NQO1 induction, CD-1 mice were fed experimental diet containing 0, 0.2, 0.3 or 0.4% DMF for 2 weeks. The mice were then killed and the liver, forestomach and kidney were removed and stored frozen at −80°C in 0.25 M sucrose until analysis for NQO1 activity.

To determine the optimal length of DMF feeding for induction of NQO1 activity, CD-1 nude mice were implanted subcutaneously in the right flanks with 5 × 10^6^ HCT116 cells and the mice were fed custom experimental diet containing no DMF for 18, 15, 11 or 4 days. The mice were then fed experimental diet containing 0.3% DMF for 0, 3, 7 or 14 days, respectively, prior to killing 18 days following tumour implantation. The HCT116 xenografts were approximately 100–250 mm^3^ at this time. The mice were killed, the organs (kidney, liver, lung, heart and forestomach) and the HCT116 xenografts were removed and marrow was obtained. All tissues were frozen at −80°C prior to measurement of NQO1 activity.

### *In vivo* combination treatment studies

For the *in vivo* combination treatment studies, CD-1 nude mice were implanted with 5 × 10^6^ HCT116 cells subcutaneously in the right flank of each mouse. The mice were fed a standard irradiated mouse diet for the first 7–10 days following tumour implantation and then randomly switched to the experimental diet containing 0 or 0.3% DMF for a further 7–10 days. At this time, day 0, mice with tumours measuring approximately 100–250 mm^3^ were randomly assigned to treatment groups. The mice were weighed and received a single tail-vein injection of saline (control diet and 0.3% DMF diet), 2.0 mg kg^−1^ MMC (control diet and 0.3% DMF diet) or 3.5 mg kg^−1^ MMC (control diet only). In addition, some mice on the 0.3% DMF diet received a single i.p injection of 34 mg kg^−1^ DIC 1 h prior to the 2.0 mg kg^−1^ MMC injection. Some mice with tumours were killed at day 0 and organs, marrow and xenografts were removed for measurement of NQO1 activity. On day 1, the diet for all the mice was switched to the standard irradiated diet. On days 0, 3, 7, 10, 15 and 18, tumour diameters in three dimensions were measured using digital calipers, and tumour volume was calculated by the formula (l × *w* × *d* × 0.5236)(Rockwell *et al*, 1972). Mouse body weights were also recorded on these same days. Differences in tumour volumes were analysed statistically by a *t*-test comparing the slopes of the regression lines for plots of tumour volume *vs* days after MMC treatment in mice receiving different treatments.

### White blood cell (WBC) and platelet counts

On days 0, 3, 6, 9, 12 and 15, approximately 20 *μ*l of blood was collected from the saphenous veins of some of the mice from each treatment group (Hem *et al*, 1998). The blood was collected into a microvette CB 300 with potassium EDTA and 10 *μ*l aliquots were used for the determination of WBC or platelet counts. The counts were determined with a Coulter Z2 particle count and cell analyser (Beckman Coulter, Mississauga, ON, Canada). The Coulter Z2 counter was optimised for mouse white blood cells and platelets according to the manufacturer's protocols and all counts were analysed with the Coulter AccuComp software program. WBC were counted with a 100 *μ*m aperture tube (set for a lower threshold of 3 *μ*m) following the addition of Zap-oglobin II lytic reagent to the coulter counter vial (50 *μ*l per 10 ml Isoton II). For counting of platelets, 10 *μ*l of mouse blood was diluted in 240 *μ*l Isoton II and spun at 83 × **g** for 3 min to remove the red blood cells. In total, 100 *μ*l of supernatant was then diluted in Isoton II and the platelets were counted using a 50 or 70 *μ*m aperture tube with a lower threshold of 1.4 fl and an upper threshold of 24.4 fl. Differences in the WBC and platelet counts in different treatment groups were analysed statistically by ANOVA for each time point.

### MMC toxicity *in vivo*

CD-1 nude mice (*n*=3) without tumours were fed a 0 or 0.3% DMF diet for 7–10 days and then were treated with a single tail-vein injection of saline (control diet), 2.0 mg kg^−1^ MMC (control diet and 0.3% DMF diet). After 7 days, the mice were anesthetised with Ketalean/Rompum and blood was obtained by cardiac puncture. The mice were then euthanised with carbon dioxide and organs were removed and fixed in neutral buffered formalin. The blood was allowed to clot and the serum was collected following centrifugation at 1500 × **g** for 15 min. The serum was stored frozen at −80°C until analysed. The serum was analysed in a Roche Hitachi 917 (Health Sciences Centre, Department of Clinical Chemistry, Winnipeg, MB, Canada) for the following parameters: Na, K, Cl, blood urea nitrogen, serum creatinine, alkaline phophatase, alanine transaminase, aspartate transaminase, gamma-glutamyl transpeptidase, lactate dehydrogenase. The organs were sectioned, stained with haematoxalin and eosin and examined histologically.

## RESULTS

### Effect of induction of NQO1 on cytotoxic activity of MMC *in vitro*

**Figure 1:**
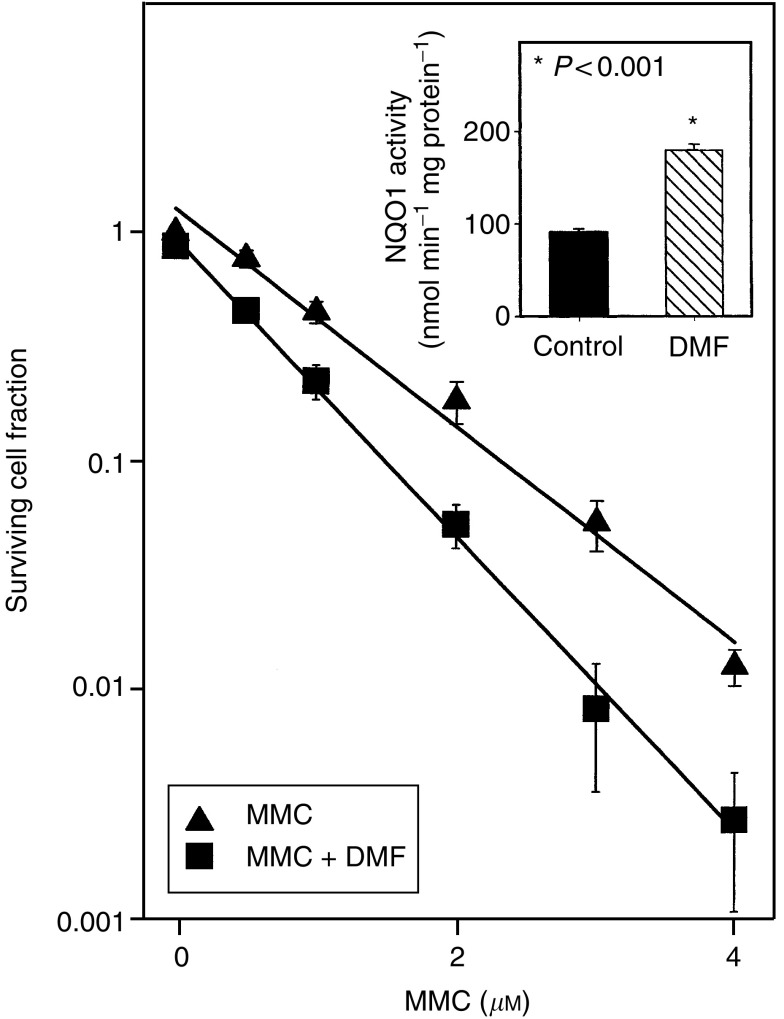
Effect of DMF on cytotoxic activity of MMC in HCT116 cells *in vitro*. Cells were incubated at 37°C for 48 h in the absence or presence of 5 *μ*M DMF. NQO1 activity was measured in some of the cells, and the remaining cells were treated with MMC for 1 h. Cells were plated and surviving cell fraction was determined by clonogenic assay. The points represent the mean surviving cell fraction±s.e. of 5–15 determinations. The lines are linear regression lines. *Inset*: level of NQO1 activity in cells incubated in the absence or presence of DMF. The bars represent the mean NQO1 activity±s.e. of 8 determinations. The means were compared by a *t*-test evaluating the significance of the difference of the NQO1 activity in control and DMF incubated cells.

When HCT116 cells were incubated *in vitro* with, or without, DMF, the level of NQO1 activity increased from 94.0±3.0 nmol min^−1^ mg^−1^ protein^−1^ in control cells to 194.0±5.3 nmol min^−1^ mg protein^−1^ in DMF treated cells (*P*<0.001) (Figure 1

). Cells that had been pretreated with DMF and then were treated with MMC were more sensitive to MMC than cells that did not receive DMF (Figure 1). The D_10_ for cells treated with MMC alone was 2.12±0.13 *μ*M while that for cells treated with DMF and MMC was 1.55±0.43 *μ*M, and this difference was statistically significant (*P*<0.001).

### Effect of dose and schedule of DMF on induction of NQO1 in HCT116 cells *in vivo*

CD-1 mice were fed a diet containing 0, 0.2, 0.3 or 0.4% DMF for 14 days. NQO1 activities in the kidney, liver and forestomach were increased in mice fed a diet containing DMF compared to mice receiving a control diet; however, enzyme activities in mice fed diet containing 0.3 or 0.4% DMF were not significantly different.

**Figure 2:**
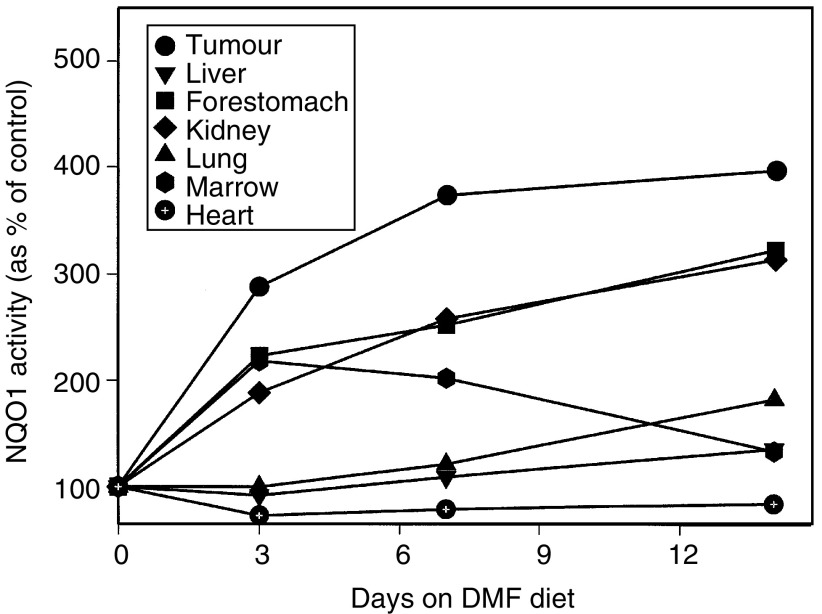
Effect of time on DMF diet on NQO1 activity in human tumour xenografts and normal tissues in CD-1 nude mice. CD-1 nude mice were implanted subcutaneously in the right flank with HCT116 cells and the mice received diet containing 0.3% DMF for 0, 3, 7 or 14 days. The mice were killed, the organs (kidney, liver, lung, heart, forestomach) and the HCT116 xenografts were removed and marrow was obtained. For each tissue, NQO1 activity was measured and is reported as the NQO1 activity in that tissue as a percent of NQO1 activity in the same tissue in mice fed diet containing no DMF. The points represent the means of values obtained from three mice.

CD-1 nude mice implanted with HCT116 cells received experimental diet containing 0.3% DMF for 0, 3, 7 or 14 days. NQO1 activity increased in the implanted tumours and reached a plateau level at approximately 7 days on the DMF diet (Figure 2

). In contrast, enzyme levels in forestomach, kidney, lung and liver tissues continued to increase to 14 days on the DMF diet, while NQO1 activity in heart tissue remained unchanged. NQO1 activity in bone marrow reached a peak at 3 days on the DMF diet, and decreased after that time.

### Effect of induction of NQO1 on MMC antitumour activity and toxicity i*n vivo*

CD-1 nude mice implanted with HCT116 cells were fed either control experimental diet or diet containing 0.3% DMF for 7–10 days. Some mice were euthanised at that time and tumours and tissues were excised. The remaining mice were weighed, tumour volumes were measured and blood samples were obtained from some mice for WBC and platelet counts. The mice were then treated with saline or DIC, and then with saline, 2.0 or 3.5 mg kg^−1^ MMC. On various days, tumour volumes were measured and blood samples were obtained for WBC and platelet counts. Average tumour volumes on the day of MMC treatment ranged from 132 to 163 mm^3^ for the different treatment groups and these differences were not statistically significant. The level of NQO1 increased by 2.5-fold in the tumours and to a similar extent in the kidneys and forestomach (*P*<0.001) in mice fed diet containing DMF (Table 1

**Table 1:** Effect of DMF diet on NQO1 activity in tumours and normal tissues

	**NQO1 activity (nmol min^−1^ mg protein^−1^)**		**Ratio**
**Tissue**	**Control diet**	**DMF diet**	**DMF/control**
Tumour	59±5	145±10	2.5*
Marrow	13±2	17±3	1.4
Kidney	135±8	329±13	2.4*
Liver	39±2	54±2	1.4*
Lung	33±5	43±4	1.3
Heart	91±5	86±7	0.9
Forestomach	362±28	952±54	2.6*

CD-1 nude mice implanted with HCT116 tumours were fed a 0 or 0.3% DMF diet for 7–10 days. Mice were euthanised and NQO1 activity was measured in different tissues. Data are mean±s.e. of 9–17 mice. NQO1 activity in mice fed control diet and diet containing DMF were compared by Mann–Whitney rank sum test.

* *P*<0.05.

). In contrast, NQO1 activity increased by 1.4-fold in liver (*P*<0.02), but did not increase significantly in bone marrow, lung or heart.

**Figure 3:**
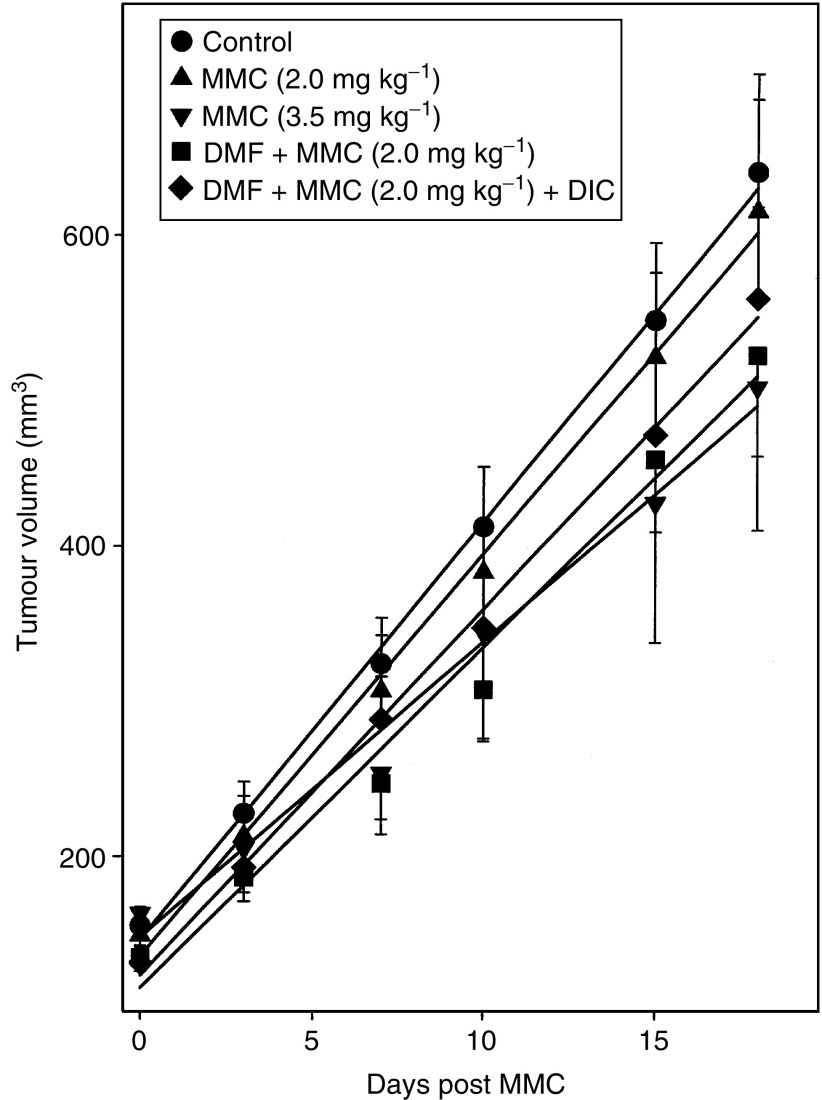
Effect of DMF diet on MMC antitumour activity in HCT116 xenografts in CD-1 nude mice. Mice were implanted with tumour cells subcutaneously in the right flank of each mouse. After 7–10 days the mice were fed a diet containing 0 or 0.3% DMF for 7–10 days. The mice were weighed and received a single tail-vein injection of saline (control diet and 0.3% DMF diet), 2.0 mg kg^−1^ MMC (control diet and 0.3% DMF diet) or 3.5 mg kg^−1^ MMC (control diet only). In addition, some mice on the 0.3% DMF diet received a single i.p. injection of 34 mg kg^−1^ DIC 1 h prior to the 2.0 mg kg^−1^ MMC injection. On days 0, 3, 7, 10, 15 and 18, tumour volumes were measured using digital calipers. The points represent the mean tumour volume±s.d. of 6–14 mice. The lines are linear regression lines.

The tumour volumes increased in all the mice, with the tumour volume in control mice (mice fed control diet and treated with saline) increasing by four-fold 18 days after treatment (Figure 3

). Mice fed control diet and treated with 2.0 mg kg^−1^ MMC showed the same rate of increase in tumour volume as control mice. In contrast, the rate of tumour growth in mice fed DMF diet and treated with 2.0 mg kg^−1^ MMC was approximately 15% lower than the rate of tumour growth in control mice or in mice fed control diet and treated with 2.0 mg kg^−1^ MMC, and this difference was statistically significant (*P*<0.01). Mice fed DMF diet that received DIC prior to 2.0 mg kg^−1^ MMC showed a partial reversal of the enhanced antitumour effect observed in mice fed DMF diet and treated with 2.0 mg kg^−1^ MMC. The antitumour effect seen in mice fed DMF diet and treated with 2.0 mg kg^−1^ MMC was equivalent to that observed in mice fed control diet and treated with 3.5 mg kg^−1^ MMC. DMF diet alone did not inhibit the increase in tumour volume (data not shown).

**Figure 4:**
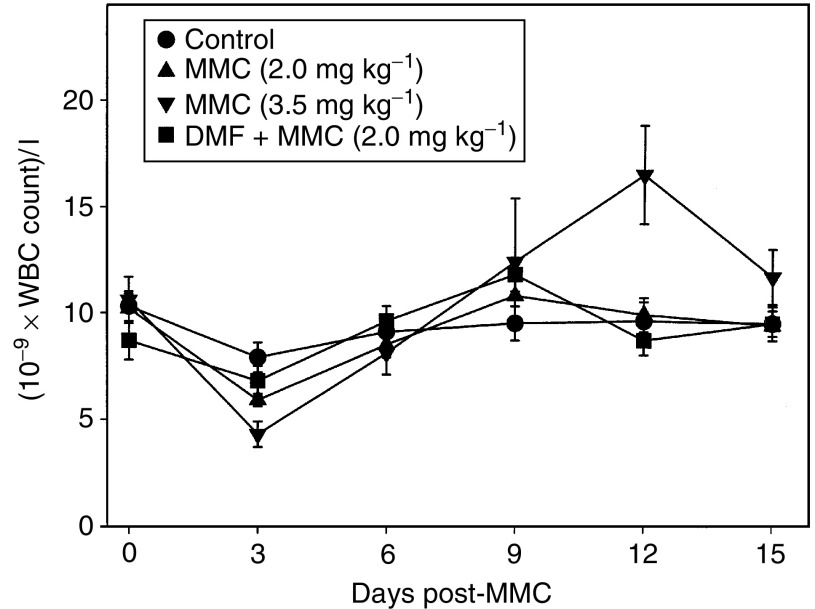
Effect of DMF diet on MMC toxicity to WBC in CD-1 nude mice. CD-1 nude mice were implanted with HCT116 cells subcutaneously in the right flank of each mouse. After 7–10 days the mice were fed diet containing 0 or 0.3% DMF for 7–10 days. The mice were weighed and received a single tail-vein injection of saline (control diet only), 2.0 mg kg^−1^ MMC (control diet and 0.3% DMF diet) or 3.5 mg kg^−1^ MMC (control diet only). On various days, approximately 20 *μ*l of blood was collected from the saphenous vein of some of the mice in each treatment group. The WBC counts were determined with a Coulter Z2 particle counter and cell analyser. The points represent the mean WBC count±s.e. of 5–16 mice. Differences in the WBC counts in different treatment groups were analysed statistically by ANOVA for each time point.

Mice fed control diet and treated with MMC showed a dose-dependent decrease in WBC counts with the nadir occurring 3 days after MMC treatment (Figure 4

). WBC counts recovered to control levels by 6 days after drug treatment. Mice fed DMF diet and treated with 2.0 mg kg^−1^ MMC showed a similar decrease in WBC counts as mice fed control diet and treated with 2.0 mg kg^−1^ MMC. DMF diet without MMC treatment had no effect on WBC count (data not shown).

**Figure 5:**
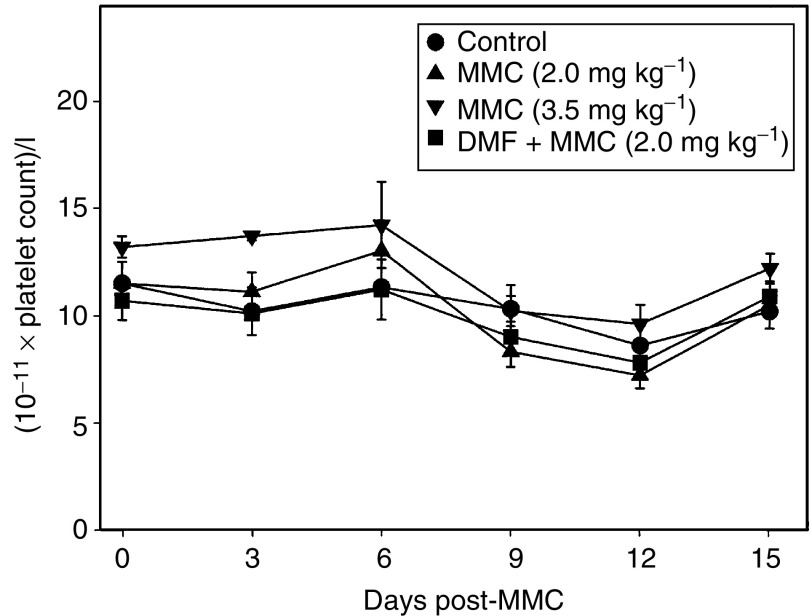
Effect of DMF diet on MMC toxicity to platelets in CD-1 nude mice. CD-1 nude mice were implanted with HCT116 cells subcutaneously in the right flank of each mouse. After 7–10 days the mice were fed a diet containing 0 or 0.3% DMF for 7–10 days. The mice were weighed and received a single tail-vein injection of saline (control diet only), 2.0 mg kg^−1^ MMC (control diet and 0.3% DMF diet) or 3.5 mg kg^−1^ MMC (control diet only). On various days approximately 20 *μ*l of blood was collected from the saphenous vein of some of the mice in each treatment group. The platelet counts were determined with a Coulter Z2 particle counter and cell analyser. The points represent the mean platelet counts±s.e. of 2–14 mice. Differences in the platelet counts in different treatment groups were analysed statistically by ANOVA for each time point.

No significant decreases in platelet counts were observed in any of the groups of mice in the 15 days following MMC treatment (Figure 5

). DMF diet without MMC treatment had no effect on platelet count (data not shown). There were no significant differences in body weight in the different treatment groups at 18 days after MMC treatment (data not shown).

In another experiment, CD-1 nude mice received either control experimental diet or diet containing 0.3% DMF for 7–10 days. The mice were then treated with saline or 2.0 mg kg^−1^ MMC and the animals were euthanised 7 days later. Histological analysis of tissues from these mice showed no evidence of damage to kidney, liver, lung, heart, colon or forestomach tissues in the MMC treated mice fed either control or DMF. Furthermore, treatment with 2.0 mg kg^−1^ MMC did not produce any significant changes in blood chemistry in mice fed control or DMF diet (Table 2

**Table 2:** Effect of DMF diet and MMC on blood chemistry

**Tissue**	**Test**	**MMC**	**DMF+MMC**
Liver	Alkaline phosphatase	Normal	Normal
	Alanine transaminase	Normal	Normal
	Aspartate transaminase	Normal	Normal
	Gamma-glutamyl transpeptidase	Normal	Normal
	Lactate dehydrogenase	Normal	Normal
Kidney	Serum creatinine	Normal	Normal
	Blood urea nitrogen	Normal	Normal
Dehydration	Sodium	Normal	Normal
	Potassium	Normal	Normal
	Chloride	Normal	Normal

CD-1 nude mice without tumours were fed a 0 or 0.3% DMF diet for 7–10 days and then were treated with a single tail-vein injection of saline (control diet), 2.0 mg kg^−1^ MMC (control diet and 0.3% DMF diet). After 7 days, three mice/group were anaesthetised and blood was obtained by cardiac puncture. The blood was allowed to clot and the serum was collected and analysed.

).

## DISCUSSION

MMC is an anticancer agent that has proved to be most effective in the front line treatment of a small number of solid tumours, such as superficial bladder, gastric, pancreatic, anal, oesophageal and non-small-cell cancer (Remers, 1979, pp 27–32; Doll *et al*, 1985; Begleiter, 2000). It is also used in palliative treatment of advanced or resistant cancers, generally in combination regimens (Begleiter, 2000). There has been continued interest in increasing the effectiveness of this agent because of its activity in solid tumours and its enhanced effectiveness against hypoxic cells that are resistant to radiation (Workman and Stratford, 1993).

Use of MMC in the clinic has been severely restricted by its toxicity (Hortobagyi, 1993; Begleiter, 2000). The usual dose-limiting toxicity is myelosuppression, which occurs 3–4 weeks after drug administration, with recovery within 8 weeks (Begleiter, 2000). Generally, thrombocytopenia or leukopenia is most severe, but anemia is also common. Less common, but potentially serious or fatal toxicities associated with MMC use include severe pulmonary toxicity that occurs in 5% of patients (Doll *et al*, 1985; Begleiter, 2000), and cancer-associated haemolytic–uremic syndrome, which can occur in 4–15% of patients (Doll *et al*, 1985; Begleiter, 2000). In addition, some cases of severe congestive heart failure have been reported after MMC treatment in patients previously treated with doxorubicin (Doll *et al*, 1985).

MMC requires intracellular activation by reductive enzymes like NQO1 and NADPH cytochrome *P*450 reductase (Rockwell *et al*, 1993; Workman and Stratford, 1993). Cells with elevated NQO1 levels are generally more sensitive to MMC (Begleiter *et al*, 1989; Marshall *et al*, 1989; Siegel *et al*, 1990), and NQO1 can be induced by a wide variety of dietary and synthetic agents (Talalay *et al*, 1988; Prestera *et al*, 1993). We have previously shown that NQO1 activity can be selectively increased in tumour cells compared with normal cells, and that this enhances MMC cytotoxicity in human and murine cell lines *in vitro* (Doherty *et al*, 1998; Wang *et al*, 1999). In this study we investigated a novel approach to increasing the antitumour activity of MMC by using a dietary component to selectively induce NQO1 activity in tumour cells compared with normal cells. We used DMF, a metabolite of fumaric acid that is found in fruits and vegetables (Talalay *et al*, 1988), as the inducer of NQO1 and HCT116 cells, which have a moderate level of NQO1 activity, as a tumour model. This approach could be used with any bioreductive agent that is activated by NQO1, but we used MMC because it is the prototype bioreductive agent and is the only bioreductive agent in widespread clinical use.

Treatment of HCT116 cells *in vitro* with DMF for 48 h increased NQO1 activity in these cells by two-fold (*P*<0.001), and cells that were pretreated with DMF were 1.4-fold more sensitive to MMC than cells that were not pretreated (*P*<0.001). This result was similar to our previous findings in murine lymphoma cells (Begleiter *et al*, 1996) and human breast, lung and colon cancer cells (Doherty *et al*, 1998; Wang *et al*, 1999) using synthetic inducers of NQO1.

To evaluate the clinical potential of this approach to increasing the efficacy of MMC, we used a human tumour xenograft mouse model to investigate whether we could obtain a similar enhancement of antitumour activity *in vivo*. This model also made it possible to evaluate any enhanced toxicity that might result from the NQO1 induction. In addition, the NQO1 inducer, DMF, was added to the diet of the mice to test the feasibility of this route of administration. In preliminary studies we found that adding DMF to the diet of mice resulted in a dose-dependent increase in NQO1 activity in various tissues; however, the increase in enzyme activity was similar with 0.3 and 0.4% DMF. Thus, 0.3% DMF was used for subsequent experiments. We also found that NQO1 activity in HCT116 tumours implanted in CD-1 nude mice reached a plateau after 7 days on the DMF diet while enzyme activities in other tissues including kidney, liver, lung and forestomach showed a further increase at 14 days. Interestingly, there was a very small increase in NQO1 activity in marrow cells that reached a peak after 3 days and declined thereafter. Therefore, for subsequent experiments, mice were fed DMF containing diet for 7–10 days prior to MMC treatment.

NQO1 activity was increased 2.5-fold in the tumours of mice fed a diet containing 0.3% DMF compared with mice fed control diet; however, there were similar increases in NQO1 activity in the kidneys and forestomachs of the inducer fed mice. In contrast, there were only small increases in NQO1 activity in the livers of DMF fed mice, and enzyme activity did not increase significantly in the bone marrow, lungs and hearts. The level of induction of NQO1 in the tumours was similar to that observed previously *in vitro*, and the lack of enzyme induction in the bone marrow was important as marrow toxicity is the dose-limiting toxicity for MMC in the clinic. The 2.5-fold increase in NQO1 in the kidneys and forestomach of the mice is of some concern due to the cancer-associated haemolytic–uremic syndrome and gastrointestinal toxicity (Doll *et al*, 1985; Begleiter, 2000) that occurs in some patients treated with MMC.

Treatment with 2.0 mg kg^−1^ MMC did not affect tumour growth in mice fed control diet. In contrast, 3.5 mg kg^−1^ MMC significantly inhibited tumour growth in mice fed control diet (*P*<0.001). However, treatment with 2.0 mg kg^−1^ MMC in mice fed DMF diet significantly inhibited tumour growth (*P*<0.01) to a level that was similar to that produced by 3.5 mg kg^−1^ MMC in mice fed control diet. Thus, the DMF diet enhanced the antitumour activity of MMC by nearly two-fold. The finding that treatment of the mice with the NQO1 inhibitor, DIC, prior to MMC partially reversed the enhanced antitumour activity in DMF fed mice strongly supports the hypothesis that the increased antitumour activity was due to the increased NQO1 activity in the tumour. This result provides strong evidence that the antitumour activity of bioreductive agents like MMC can be enhanced by induction of NQO1 *in vivo*.

MMC doses of 2.0 and 3.5 mg kg^−1^ MMC decreased WBC counts by 25 and 45%, respectively, 3 days after drug treatment in control fed mice, but the counts recovered approximately 6 days after treatment. There was a further increase in WBC counts in the mice treated with 3.5 mg kg^−1^ that reached 150% of normal levels 12 days after MMC treatment, but the counts returned to normal 3 days later. These results are similar to findings reported previously for the effect of MMC treatment in mice (Shimomura *et al*, 1988). WBC counts decreased 3 days after treatment in mice fed DMF diet and treated with 2.0 mg kg^−1^, but this effect was not greater than that seen in mice fed control diet and receiving 2.0 mg kg^−1^ MMC. In contrast, while there was some variation with time, we did not observe any significant decrease in platelet counts in any of the treatment groups. This result differs somewhat from previous studies that found a decrease in platelet counts in mice treated with MMC with the nadir occurring at approximately 7 days following drug treatment (Shimomura *et al*, 1988). This difference may be due to the relatively low dose of MMC used in this study or to differences in the type of mice used. Overall, these results demonstrated that induction of NQO1 with DMF did not increase MMC marrow toxicity.

We found no significant weight loss in mice treated with 2.0 mg kg^−1^ MMC whether they were fed control or DMF diet. In addition, there was no histological evidence of damage to kidney, liver, lung, heart, colon or forestomach tissue in MMC treated mice that were fed either control or DMF diet. Furthermore, blood chemistry was normal in mice in all the treatment groups indicating no damage to the kidneys or livers. This was true despite the increase in NQO1 activity observed in kidney and forestomach tissues. This lack of effect may reflect the dose of MMC used, but demonstrates that induction of NQO1 does not result in toxicity when combined with subtoxic doses of MMC.

To examine the limits of these findings we treated some DMF fed mice with 3.5 mg kg^−1^ MMC (data not shown). We saw almost no tumour growth in these mice, but we did observed a delayed weight loss in some of the mice starting at approximately 8 days after MMC treatment. However, there was no evidence of any damage to the kidneys, livers, lungs, hearts, forestomach or the entire gastrointestinal tract by histological examination, or changes in blood chemistry. Nor did we observe any changes in food consumption or changes in sodium or potassium levels that would indicate dehydration in the mice. The cause of the observed weight loss in these mice remains unknown. There have been some reports of anorexia associated with MMC use in the clinic, but the cause of this effect is also unknown. Thus, it is not clear whether the weight loss we observed in the DMF fed mice receiving 3.5 mg kg^−1^ MMC would be restricted to mice or would also be a concern in humans. In addition, it should be noted that the potential toxic effects of combining DMF diet with MMC were studied in a mouse model, while the antitumour effects were studied in human tumours. Although we have previously shown that induction of NQO1 in human and murine tumours and in human and murine bone marrow cells is similar (Begleiter *et al*, 1996; Doherty *et al*, 1998), induction of NQO1 in other human tissues has not been extensively studied. This suggests that caution should be used in applying this approach to enhancing the antitumour activity of MMC in the clinic.

These studies demonstrated that dietary inducers can be used to selectively increase the level of NQO1 activity in tumours. Combining induction of NQO1 with treatment using a bioreductive antitumour agent that is activated by NQO1 can significantly enhance the antitumour activity of the agent *in vivo* without increasing toxicity. In the case of MMC, this approach might best be used by combining an NQO1 inducer with a reduced dose of MMC. This would produce an antitumour effect that was equivalent to the normal dose of MMC with reduced toxicity, especially myelosuppression, resulting in a faster recovery time and allowing more frequent retreatment. The use of a dietary component to induce NQO1 would also facilitate clinical evaluation of this strategy. In this study, we observed a two-fold enhancement of MMC antitumour activity. While this effect is relatively small, it could have a significant clinical impact if the combination therapy were used as described above. However, NQO1 is not the most important activating enzyme for MMC and may contribute only approximately 30% to its activation (Rockwell *et al*, 1993). Thus, induction of NQO1 might produce a greater enhancement of antitumour activity, and an even greater clinical effect, when combined with a bioreductive agent like RH1, which is selectively activated by NQO1 (Winski *et al*, 1998). Studies to investigate this possibility are in progress.
